# Unroofed Coronary Sinus Presenting as Cerebral Abscess: A Case Report

**DOI:** 10.4021/cr273w

**Published:** 2013-07-11

**Authors:** Avinash Murthy, Ankit Jain, Mohammad El-Hajjar

**Affiliations:** a Division of Cardiology, Albany Medical Center, MC-44, 47 New Scotland Avenue, Albany, NY, 12208, USA

**Keywords:** Coronary anomalies, ALCAPA, Sudden cardiac death, Congenital heart disease

## Abstract

A sixty eight year-old woman with a long-standing history of hypertension, dizziness and a history of congenital heart disease presented with speech difficulties and disorientation. She was diagnosed with a brain abscess, confirmed by a stereotactic biopsy. Transthoracic echocardiographic evaluation revealed a persistent left superior vena cava (PLSVC) with an unroofed coronary sinus (URCS) along with a small secundum atrial septal defect. Her heart catheterization showed a partially unroofed coronary sinus along with a bidirectional shunt. She was referred for surgical closure of her unroofed coronary sinus and the secundum atrial septal defect. Her brain abscess responded well to antibiotic treatment. While waiting for open-heart surgery, she suffered from an acute myocardial infarction and underwent emergent percutaneous coronary intervention to the right coronary artery. Subsequently, she underwent elective surgical repair of the unroofed coronary sinus, along with closure of the atrial septal defect. When she was seen in follow-up she reported a complete resolution of her dizziness and felt more energetic. Unroofed coronary sinus syndrome (URCS) is a rare congenital cardiac anomaly in which there is a communication between the coronary sinus and the left atrium. While non-invasive imaging with echocardiography, MRI or CT is helpful in making the diagnosis, cardiac catheterization remains integral in the evaluation and management planning. Management is guided by the presence of clinical symptoms with consideration of repair when patients become symptomatic. Prognosis after surgery is excellent, recently transcatheter based treatment therapies are becoming more frequent. We present a rare case of URCS with PLSVC presenting as a cerebral abscess in late adulthood. She had bidirectional shunting manifesting as a cerebral abscess. She responded well to the corrective surgery and was doing well on follow up.

## Introduction

Unroofed coronary sinus syndrome (URCS) is a rare congenital cardiac disorder and has a strong association with a persistent left superior vena cava (PLSVC). Patients may be asymptomatic or may have a variety of symptoms. Non-invasive cardiac imaging is useful to make the diagnosis. A high degree of suspicion facilitates early diagnosis. Management is usually guided by the presenting clinical symptoms. Prognosis after surgery is excellent. Recently, transcatheter based treatment therapies are increasingly used to correct this condition.

## Case Report

**Figure 1:**
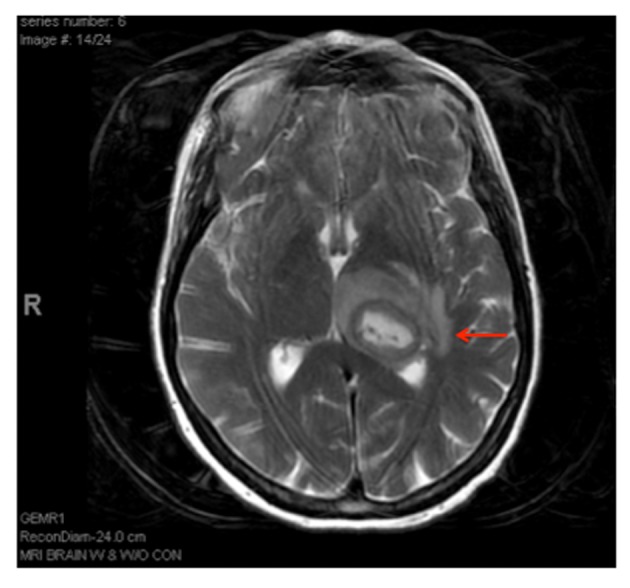
Necrotic left thalamic rim enhancing mass measuring about 2.5 × 2.3 cm.

**Figure 2:**
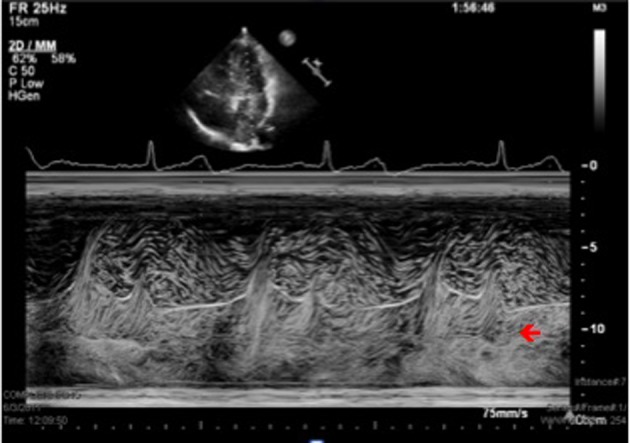
M Mode Echocardiography through the left atrium and left ventricle showing bubbles after agitated saline injection in the left arm.

**Figure 3:**
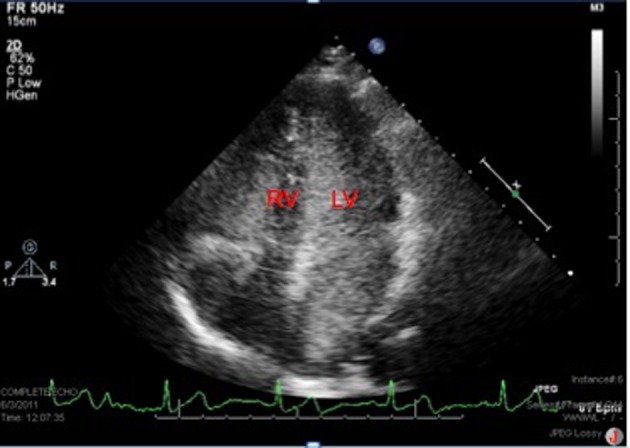
The 2 D echocardiogram showing bubbles predominantly in the left atrium and left ventricle after agitated saline injection in the left arm, note few bubbles in the right heart as well due to the ASD and left to right shunt.

**Figure 4:**
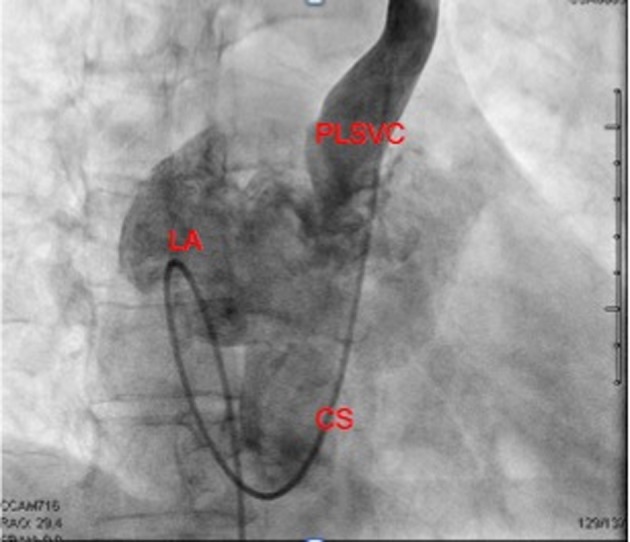
RAO Cranial View on biplanar angiography showing the catheter via a femoral approach entering the right atrium, then through the unroofed coronary sinus into the left atrium and the PSLVC. Selective dye injection into the PLSVC opacifies the left atrium and the coronary sinus.

**Figure 5:**
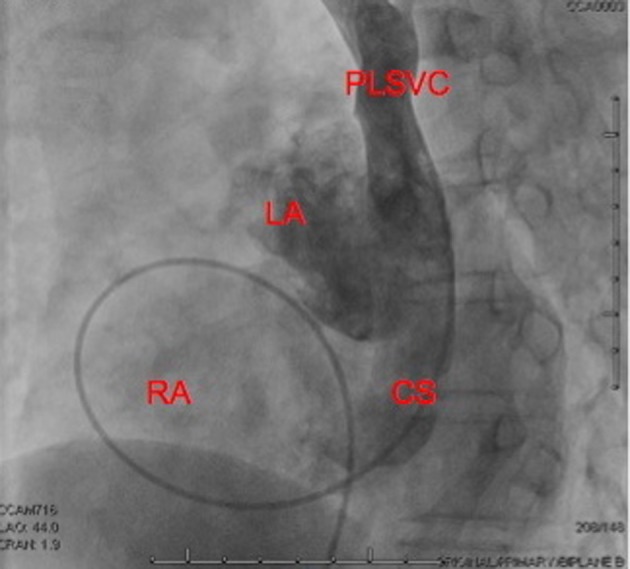
LAO Cranial View on biplanar angiography showing the left atrium posterior to the right atrium. Selective dye injection into the PLSVC opacifies the CS and the left atrium.

A sixty eight year-old woman with a long-standing history of hypertension, dizziness presented with confusion, aphasia and right-sided weakness. She reported some congenital heart condition (however there were no operation scars on the chest wall) without any cyanosis. Imaging of the brain revealed a 2 × 2.3 cm cystic structure in the left thalamus (Fig. 1). She was diagnosed with a brain abscess, which was surgically drained and treated with a full course of antibiotics. The patient had a stereotactic biopsy of the left thalamic mass and placement of ventriculostomy. The biopsy results were suggestive of an abscess with Streptococcus intermedius and Eikenella corrodens. She was started on six weeks of IV antibiotics. Due to the embolic nature of the abscess, she was referred for an echocardiographic evaluation. With bubbles injected from the left arm, the transthoracic echocardiogram revealed a persistent left superior vena cava (PLSVC) draining into left atrium consistent with an unroofed coronary sinus (URCS) along with a small secundum atrial septal defect (Fig. 2, 3). During her heart catheterization, a left superior vena cava venography was performed for further assessment. Her left brachiocephalic vein drained through a persistent left superior vena cava into a dilated coronary sinus. The coronary sinus was partially unroofed in the proximal to mid portion with a large opening into the upper side of the left atrium before it continued to drain in the right atrium. Serial blood samples for oxygen saturation were obtained at the level of the superior vena cava, inferior vena cava, right atrium, right pulmonary vein and the pulmonary artery. The study revealed bidirectional shunting through the unroofed coronary sinus between the left atrium and right atrium (Fig. 4, 5). Using the Fick method, oxygen consumption of 125 mL/min/m^2^, and hemoglobin of 10 g/dL, we calculated the pulmonary flow (QP) at 5.9 L/min, the systemic flow (QS) at 4.5 L/min, and the effective cardiac output (Qeff) at 3.9 L/min. The right-to-left shunt was 0.6 L/min, the QS/Qeff was 1.15, the left-to-right shunt was 2 L/min, and the QP/Qeff was 1.5. It was also noted that the patient’s finger oxygen saturation was fluctuating between 83% and 91% during the procedure, reflecting variation in the magnitude of her right-to-left shunt. Her mean right atrium pressure was 9 mmHg, right ventricle systolic pressure was 35 mmHg with an end-diastolic of 11 mmHg, and pulmonary artery pressure was 37/12 mmHg with a mean of 22 mmHg. She did not have any obstructive coronary artery disease. She was diagnosed with an URCS with PLSVC. In light of the brain abscess and bidirectional shunting, she was referred for surgical closure of her unroofed coronary sinus and the secundum atrial septal defect. While waiting for open-heart surgery as an outpatient, unfortunately she suffered from an acute myocardial infarction and underwent emergent percutaneous coronary intervention to the right coronary artery. Subsequently, she underwent elective surgical repair of the unroofed coronary sinus, along with closure of the atrial septal defect, using pericardial patch and gortex graft material. She had an excellent recovery from her heart surgery. During follow up she surprisingly expressed a complete resolution of her dizziness and felt more energetic as compared to what she was used to in her lifetime. Our patient illustrates a rare case of URCS with PLSVC presenting as a paradoxical septic embolus to the brain in late adulthood. Surgical repair of her condition proved worthy as evidenced by a dramatic improvement in her long-standing symptomatology.

## Discussion

Unroofed coronary sinus syndrome (URCS) is a rare congenital cardiac anomaly in which there is a communication between the coronary sinus and the left atrium as a result of the partial or complete absence of the roof of the coronary sinus. It is the most rare form of an atrial septal defect. It has a strong association with a persistent left superior vena cava (PLSVC), with or without a connection between both superior venae cavae [1]. URCS comprises less than 1% of all types of atrial septal defects and can also be associated with other forms of other congenital heart abnormalities like canal defects, cor triatrium, tetralogy of Fallot, pulmonary atresia or stenosis, abnormal ventriculo-arterial connection, and anomalous pulmonary venous return [2]. PLSVC remains the most common association. In the series described by Quaegebeur et al [3], 75% of cases of URCS were associated with PLSVC.

Normally during early development, the anterior cardinal veins unite with the posterior cardinal veins and enter the heart as the right and left horns of sinus venous along with the other veins. After development of the innominate vein, the left cardinal vein slowly obliterates with the remnant known as the ligament of Marshall while the proximal portion of the left sinus horn develops into the coronary sinus. Failure of the obliteration of the left anterior cardinal vein results in PLSVC. PLSVC is seen in 0.3% of the general population and it usually drains into the right atrium via the coronary sinus. In about 8% of people with PLSVC, the PLSVC drains into the left atrium [4].

When PLSVC drains into the coronary sinus, which lies in the posterior interventricular groove, it causes an enlargement of the coronary sinus. Improper formation of the posterior wall of the left atrium results in URCS or a coronary sinus type ASD [4]. This best explains the increased association between the PLSVC and the unroofed coronary sinus. Alternative theory stating dissolution of the partition between coronary sinus and left atrium resulting in URCS had been put forth [5]. Kirklin and Barratt- Boyes have classified unroofed coronary sinus morphologically into four types [1]: type 1, completely unroofed with PLSVC; type 2, completely unroofed without PLSVC; type 3, partially unroofed mid portion; and type 4, partially unroofed terminal portion.

In the absence of bridging veins between the venae cava, patients with isolated PLSVC drain into the left atrium results in a right to left shunt [6]. These patients have mild cyanosis and polycythemia. Hence isolated PLSVC is often an incidental diagnosis. However, in the presence of URCS, the patients may be asymptomatic or may present with cyanosis, right heart failure, or due to complications of paradoxical embolism via a right to left shunt [7]. The diversion of systemic venous blood into the left atrium is more prominent when there is atresia, stenosis or hypoplasia of the coronary sinus ostium [8]. Left to right shunt has been noted when an unroofed coronary sinus is present in isolation, or together with an uncomplicated atrial septal defect [9]. Bidirectional atrial shunting has also been reported. The clinical presentation depends on the size of the defect and the degree of shunt [10] which in turn is determined by the right and left ventricular compliance. Nonetheless the diagnosis remains challenging because the clinical signs and symptoms are not specific [11]. Entities like right heart enlargement, paradoxical embolism, transient hypoxia, or cyanosis should raise a suspicion and the diagnosis of URCS should be considered [10].

Transthoracic echocardiography is the most widely used noninvasive study technique, however, posterior structures like pulmonary veins or coronary sinus may not be well seen. Contrast studies with agitated saline injection in the left arm showing bubbles in the coronary sinus prior to its arrival in the right atrium indicate PLSVC. More recently 3D echocardiography has been used to identify and delineate the anatomy [12]. Transesophogeal echocardiography is useful to evaluate the pulmonary veins and the coronary sinus. The spin-echo and cine magnetic resonance imaging (MRI) and the multidetector computer tomography (MDCT) provide excellent anatomical evaluation. Phase-contrast velocity-encoded cine MRI can be used to calculate the shunt fraction [13]. Such detailed imaging helps in planning for surgery by providing detailed 3D anatomical information and by detecting any co existing cardiac lesion [14]. One can expect an increasing incidence as we do more and more MDCT and MRI imaging studies for various reasons and incidentally find URCS with or without PLSVC. Cardiac catheterization remains integral in the evaluation by defining the shunt direction, quantifying the shunt fraction, diagnosing pulmonary hypertension, detecting other valvular diseases and coronary artery disease while delineating the anatomy. It can be challenging and catheterization via the left arm is often required. Diagnosis is made usually by selective injection of contrast material in the PLSVC or coronary sinus. The catheter is inserted from the right atrium to the left atrium via the atrial septal defect or coronary sinus. Occasionally as in our case, the catheter is advanced further in the coronary sinus into the PLSVC. Catheter insertion to the coronary sinus rarely can cause chest pain or arrhythmia [15].

Complications of URCS with a PLSVC include pulmonary hypertension due to chronic left to right shunt and increased risk of brain abscess or systemic emboli when it progresses to a right to left shunt [16]. In undiagnosed PLSVC, catheterization of the left internal jugular or subclavian vein may lead to aberrant pulmonary artery trajectory [17]. Aberrant left heart and aortic catheterization, is also possible, resulting in vascular injuries, embolism, spurious pulmonary hypertension amongst others [18].

Management is guided by the presence of clinical symptoms with consideration of repair when symptoms prevail. Watanabe et al. reported spontaneous closure of an unroofed coronary sinus in 2004 in a one-day-old boy following emergency balloon atrioseptostomy & Blalock-Hanlon procedure [19]. Transcatheter based treatment therapies are becoming more frequent due to its relative ease as compared to open-heart surgeries. PLSVC has been successfully occluded using Greenfield vene cava filter and coils. One must ensure that there are enough collateral veins between the right SVC and PLSVC to enable venous return prior to PLSVC occlusion [20]. More recently URCS has been successfully treated using a covered stent [21] and by implantation of an Amplatzer septal occluder device [22]. There is more experience with surgical repair, which mainly involves rerouting of the PLSVC blood flow, with or without preservation of the PLSVC along with correction of the atrial septal defect. Prognosis after surgery is excellent. In the study by Ootaki et al, follow up for an average of 85.5 months in 11 patients after surgical repair revealed no deaths or complications related to surgery [16].

To the best of our knowledge, this case of URCS with PLSVC is the only one reported to present as a cerebral abscess in late adulthood. The patient had bidirectional shunting manifested as dizziness, as a cerebral abscess. Echocardiography with bubble injection from the left arm established the diagnosis. Cardiac catheterization provided all necessary information needed for her surgical management. Corrective surgery late in life proved worthy and resulted in complete relief of her chronic symptoms. It is difficult to diagnose this entity; a high-index of suspicion is required in select cases for early diagnosis and successful management.
